# Fecal microbiota transplantation for multiple organ dysfunction syndrome

**DOI:** 10.1186/s13054-016-1567-z

**Published:** 2016-12-19

**Authors:** Nathan J. Klingensmith, Craig M. Coopersmith

**Affiliations:** 1 Department of Surgery and Emory Critical Care Center, Emory University School of Medicine, Atlanta, GA USA; 2 Emory University School of Medicine, 101 Woodruff Circle, Suite WMB 5105, Atlanta, GA 30322 USA

**Keywords:** Sepsis, Fecal microbial transplantation, MODS, Diarrhea, Therapeutic efficacy, 16s rRNA, Firmicutes, Proteobacteria

Approximately 40 trillion bacteria reside inside the human intestine, meaning there are at least as many cells of microbial origin as human origin [1]. While it was once believed that bacteria and humans simply co-existed in the same space, a wide body of evidence now suggests that host–microbial communication is more complex than ever imagined and the microbiome plays a critical role in maintaining host homeostasis. The microbiome is also altered in multiple disease states, including heart disease [2], cancer [3], and *Clostridium difficile* infection [4], with changes detectable in microbial composition, number, diversity, and virulence compared to healthy controls. While the majority of studies linking the microbiome to disease are associative, there is increasing evidence that the microbiome plays a crucial role in mediating the pathophysiology of multiple acute and chronic illnesses.

The gut has long been hypothesized to be “the motor” of multiple organ dysfunction syndrome (MODS) [5]. Notably, the microbiome is markedly altered in the intensive care unit (ICU). A recent study sampling stool from 115 critically ill patients revealed decreases in *Firmicutes* and *Bacteroidetes—*bacteria that are prevalent in the healthy intestine—with increases in opportunistic *Proteobacteria* [6]. Although these findings cannot determine whether an altered microbiome functions as a marker or mediator in the development of MODS, it raises the question as to whether altering microbial ecology and restoring diversity of the microbiome may be a novel therapeutic strategy in critical illness.

Multiple methods to manipulate host bacteria have been tried in patients, ranging from giving exogenous bacteria in the form of probiotics to selecting out pathogenic microbes via selective decontamination of the digestive tract. While each of these strategies has shown some clinical promise [7, 8], debatably the most effective method of altering the microbiome in human disease is fecal microbial transplantation (FMT). FMT is a procedure where stool is collected from a healthy donor, filtered for particulate matter, and the liquid portion given to the patient via nasogastric tube or via the rectum [9]. FMT has been demonstrated to be significantly more effective in recurrent *C. difficile* infections than other available therapies [10]. Treatment failures still occur, however, which is not surprising given that we do not yet fully understand the mechanisms responsible for the success of FMT, nor do we know the optimal dose and timing required for a successful transplant. Notably, viruses, fungi, viable colonocytes, immunoglobulins, metabolites, and natural bacteriocins may be present in FMT and impact outcomes [11, 12].

The literature on FMT in critical care is limited to a single case report [11]. The barriers to utilizing FMT in the ICU are significant. The impact of giving bacteria to a patient with a markedly altered microbiome who likely has a component of immunosuppression is unknown and has inherent theoretical risks. In addition, the majority of critically ill patients receive antibiotics at some point during their ICU stay, and initiation or continuation of antibiotic therapy would be expected to markedly alter the microbiome following FMT. As such, there must be a commitment to stopping antibiotics both prior to and following FMT to allow the transplanted bacteria to take hold and remodel the microbiome. This is a difficult action to take unless it is clear that a patient is not infected, a clinical judgment which unfortunately is not always straightforward at the bedside.

Wei et al. recently reported the use of FMT in two critically ill patients who developed sepsis during their hospital course [13]. Each was successfully treated with multiple antibiotics (which sterilized their cultures) but was left with MODS and non-*C. difficile* diarrhea, refractory to standard medical management. Analysis of stool from both patients demonstrated marked alterations in the microbiome compared to healthy patients. The investigators concluded that the patients suffered from intestinal dysbiosis not related to active infection and would therefore be appropriate candidates for FMT. Antibiotics were held, and three days later FMT was performed. Over the 2–3 weeks following FMT, both patients had resolution of their diarrhea and improvement in multiple markers of inflammation such as interleukin-6, C reactive protein, procalcitonin, and erythrocyte sedimentation rate. Notably, the investigators followed the bacterial composition of the patients’ stool using 16s rRNA pyrosequencing, both immediately before and up to 20 days after FMT. Following FMT, the composition and diversity in both patients shifted toward that of the donor with increasing *Firmicutes* and decreasing *Proteobacteria*.

Outside of antibiotics, supportive care remains the mainstay of treatment in the ICU [14], and any new therapy that has the capacity to cure a condition in critical illness is potentially exciting. However, the efficacy seen with FMT in this study must be interpreted with caution. First, while a rich and diverse intestinal microbiome is associated with health [15], this does not inherently mean that the altered microbiome plays a causative role in MODS or that restoration to a normal state in critical illness is beneficial. In the cases presented here, stool volume and inflammatory markers were already decreasing prior to initiation of FMT, and it is possible the patients would have recovered without receiving this experimental therapy. Assuming FMT was indeed responsible for the ultimate improvement in patient course, the degree of generalizability of the results presented is unclear, as with any case report. For instance, if one imagines a clinical trial of FMT that mimics the patients studied, the entry criteria would be relatively narrow—patients with MODS and dysbiosis-induced non-infectious diarrhea who do not require antibiotics. As such, FMT must be considered experimental in critical illness until rigorous trials are conducted. Nonetheless, the opportunity to manipulate our microbiome for therapeutic benefit in the ICU represents a tantalizing new direction in the future care of critically ill patients.

## Abbreviations

FMT: Fecal microbial transplantation
ICU: intensive care unit
MODS: multiple organ dysfunction syndrome.
